# Mutualism in museums: A model for engaging undergraduates in biodiversity science

**DOI:** 10.1371/journal.pbio.2003318

**Published:** 2017-11-21

**Authors:** Anna E. Hiller, Carla Cicero, Monica J. Albe, Theresa L. W. Barclay, Carol L. Spencer, Michelle S. Koo, Rauri C. K. Bowie, Eileen A. Lacey

**Affiliations:** 1 Museum of Vertebrate Zoology, University of California, Berkeley, California, United States of America; 2 Department of Integrative Biology, University of California, Berkeley, California, United States of America

## Abstract

Museums have an untapped potential to engage students in hands-on learning. Here, we describe the development of a tiered museum-based program at the University of California, Berkeley as a model for engaging undergraduates in biodiversity science. This decade-long effort to increase student participation in collections demonstrates the mutual benefits of undergraduate involvement. Museums benefit from critical help in collections care and an increased intellectual vitality, while students simultaneously gain essential research skills and an unparalleled exposure to biodiversity. Five first steps to creating a program are: dedicate a coordinator, offer credit, diversify participation, create a tiered structure, and build community.

## Introduction

Natural history museums have an incredible but largely untapped potential to facilitate undergraduate learning through specimen-based research and teaching [1,2]. Although best known for their value as historical “libraries of life” [3,4], biodiversity collections are also powerful educational tools [1,5]. Particularly crucial is the role that natural history museums can play in training future generations of research scientists, conservationists, and professionals.

The past few decades have witnessed a tendency to de-emphasize undergraduate training in natural history, biodiversity science, and field biology [6,7]. This trend reflects multiple factors, including funding cuts and increased regulatory oversight. However, we believe such challenges can be overcome through educational initiatives like the Advancing Integration of Museums into Undergraduate Programs (AIM-UP!) course modules [1], Course-Based Undergraduate Research Experiences (CUREs) [8], and the program we describe here.

Undergraduates in museums have the opportunity to see and hold biodiversity in their hands. This kind of hands-on learning imparts students with an exceptionally deep understanding of ecology and evolution [9,10]. In addition, these students gain broad exposure to both organismal research and practical skills such as data management. Benefits are not one-sided, however. In the current era of staff reductions [11,12], funding cuts [13], and infrastructure declines [3,14,15], museums have much to gain by involving undergraduates as a supplementary form of personnel. Equally important, students provide significant intellectual contributions to museums by bringing new perspectives and innovations. The mutualistic benefits of engaging students in museums can lead to exciting new opportunities in biodiversity science.

Here, we present an example of an exceptionally effective and in-depth program that incorporates active, hands-on learning in undergraduate education: the Undergraduate Program at the Museum of Vertebrate Zoology (MVZ), University of California, Berkeley. We detail the program’s structure as a model for engaging students in biodiversity science and conclude with several recommendations for other museums interested in developing similar programs.

## Overview of program structure

**Fig 1:**
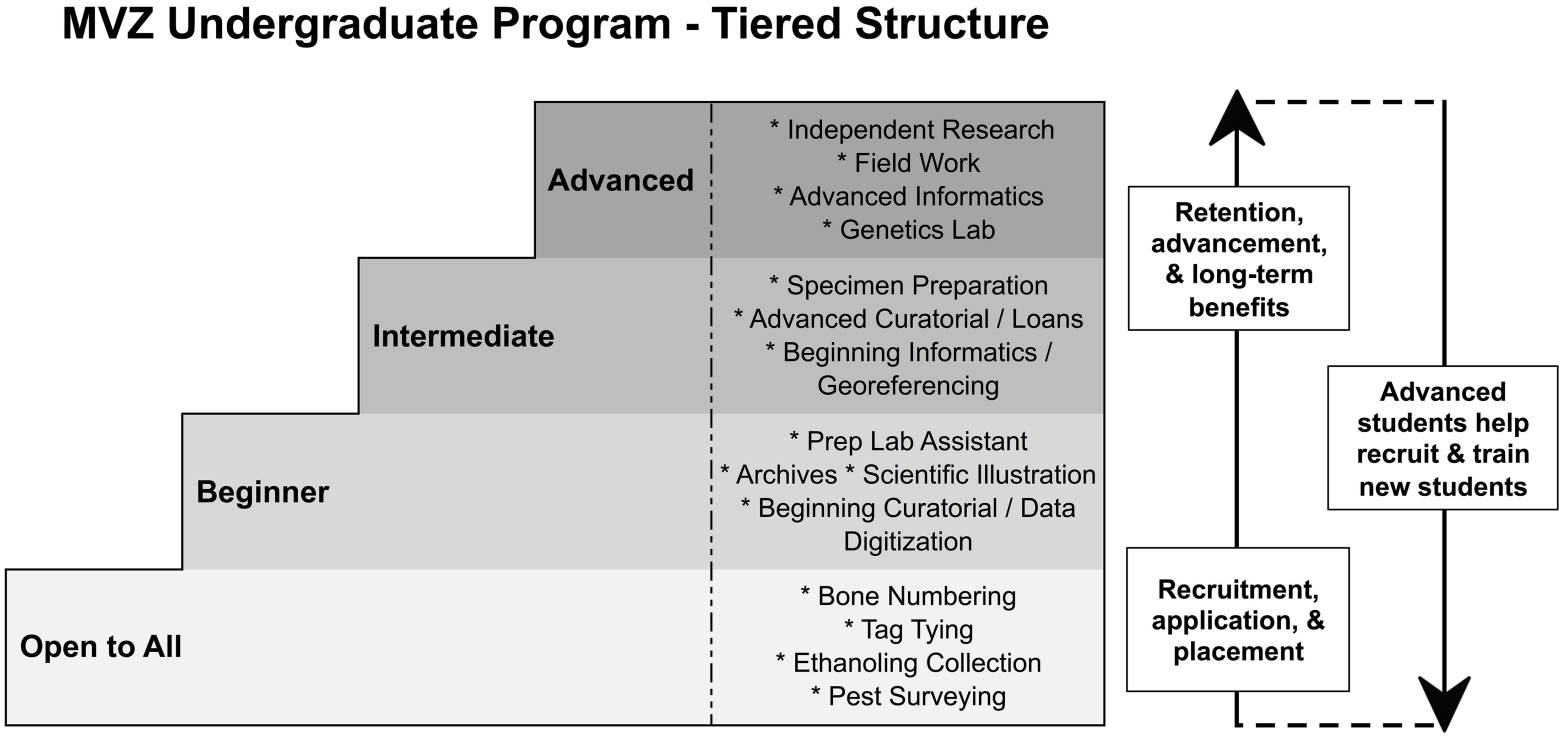
Tiered structure. The stepwise progression of the MVZ Undergraduate Program allows students with no previous experience (e.g., “Open to All” and “Beginner”) to join the MVZ and then advance to more complex positions (e.g., “Intermediate” and “Advanced”) as they gain experience. Advanced students also give back to the program by helping to recruit and train new students. MVZ, Museum of Vertebrate Zoology.

**Fig 2:**
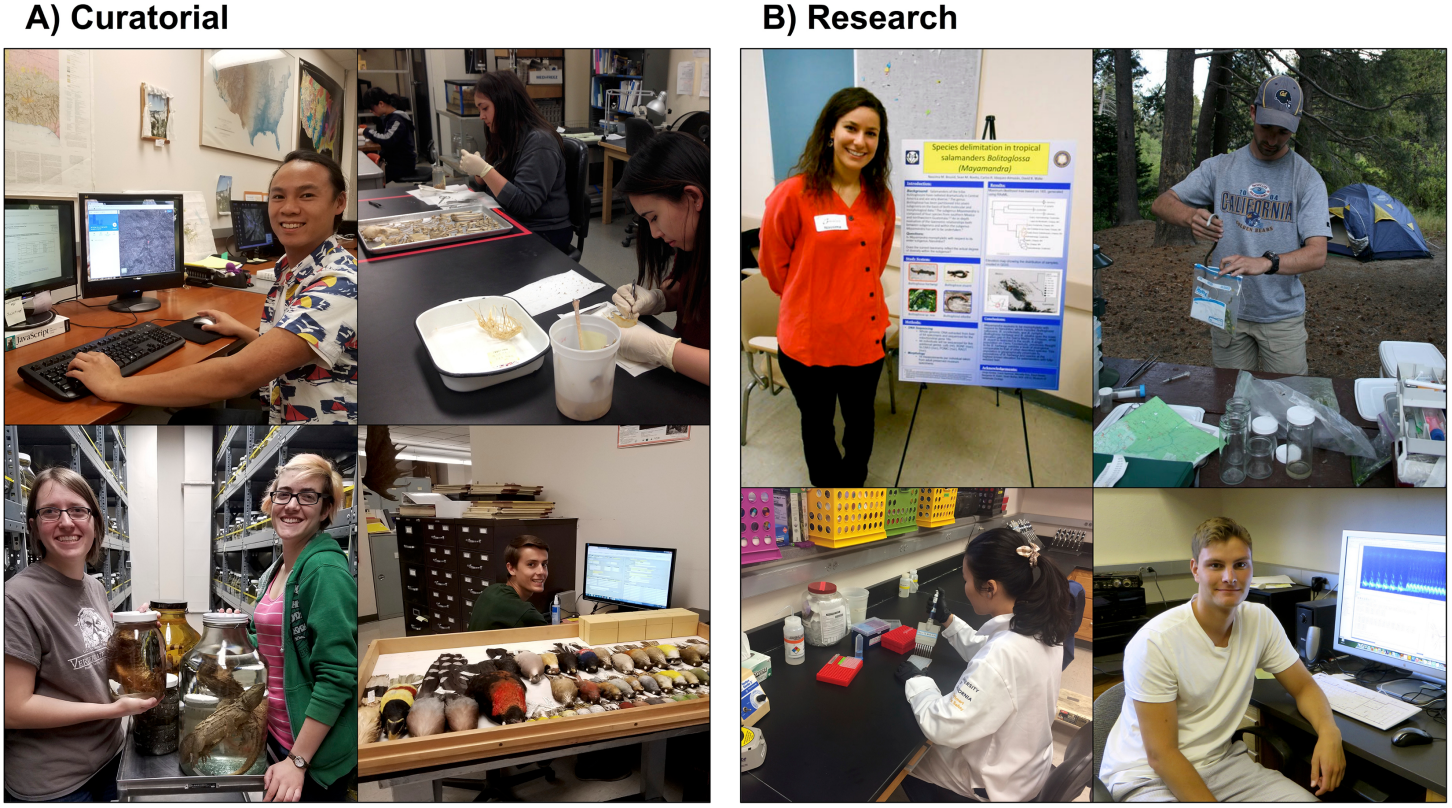
Undergraduates engaged in active-learning. **(A)** Members of the MVZ Undergraduate Program involved in curatorial and specimen preparation activities. Clockwise from top left: georeferencing specimen localities, cleaning skeletal material, cataloging bird specimens, and topping off ethanol in jars of fluid specimens. **(B)** Members of the MVZ Undergraduate Program involved in museum-based research projects. Clockwise from top left: presenting research at a conference, collecting herpetological specimens in the Sierra Nevada, analyzing digital recordings of bird songs, and conducting molecular research in the Evolutionary Genetics Lab. MVZ, Museum of Vertebrate Zoology.

Use of a tiered structure (Fig 1) allows students to advance as they gain training and experience. Curatorial responsibilities (Fig 2) range from introductory tasks (e.g., numbering bones) to higher-level work (e.g., cataloging, georeferencing). All of these activities involve interacting with specimens, thereby imparting students with an appreciation for biodiversity and collections. As they progress, students can participate directly in specimen-based research (e.g., genomics, morphometrics, bioacoustics, field work; Fig 2), which comprises the most intellectually sophisticated form of undergraduate involvement.

The MVZ program is open to all UC Berkeley students, who are recruited via multiple routes. This increases the applicant pool and attracts individuals with diverse backgrounds and interests (S1 Fig). Students most commonly enter the program through Berkeley’s Undergraduate Research Apprentice Program (URAP). These students receive academic units for time spent on a project, recorded as “Biological Research” on their transcripts. Applicants are interviewed and then placed in positions that best suit their interests and skill levels (S2 Text).

At the end of each semester, students and supervisors provide feedback that is used both to evaluate the program and to advance undergraduates through the tiered system (Fig 1). More experienced students help to train incoming undergraduates, which distributes teaching and mentorship duties and contributes to the sense of progression through the program. Step-by-step manuals also have been developed to minimize training time for certain tasks. Today, the MVZ hosts one of the largest undergraduate programs offered at any natural history museum, with over 100 students per semester. This quantity of students helps to sustain and enrich the program by creating a “critical mass” of undergraduates at different stages in their education.

## Key program elements

The MVZ is not unique among museums in engaging undergraduates in curatorial and research activities [1]. However, at least four factors make this program design especially successful and comprehensive.

### Links to formal education

Undergraduates often take courses in biodiversity science that complement and enrich the program. A freshman seminar introduces new students to the collections and museum research opportunities. Many classes use specimens in lab exercises and incorporate field trips during which students connect what they’ve learned in the museum with vertebrates in their natural settings. For example, the century-old “Natural History of the Vertebrates” course at UC Berkeley [16] has weekly labs and field trips to local regional parks. By working in the museum and also taking this class, students reinforce what they learn about species identification, data collection, and recording natural history notes. For museums that are not affiliated with universities, we suggest that workshops or speaker-led seminars could provide the same kinds of instruction.

### Collection accessibility

The museum’s central location on the Berkeley campus makes it easier to involve students because they can work in the collections between classes. In an era when multiple university collections have been eliminated or moved offsite [17], the large population of undergraduates has also helped to ensure continued university support for the museum and to protect its presence on an extremely space-constrained campus. For institutions not centrally located, making collections accessible will be critical to promoting undergraduate participation.

### Specimen preparation

One of the most popular opportunities in the program is to work as a preparation lab assistant (Fig 2) or to take one of the museum’s two specimen preparation classes, where students receive direct, hands-on exposure to vertebrate diversity via dissections. Students are first trained to prepare taxonomically diverse skeletons and then expand their skills by preparing study skins of birds or mammals. In the process, they learn to identify species using field guides, to document biological attributes (e.g., sex, age, reproductive status), and to appreciate the value of meticulous data collection. Advanced undergraduates lead these classes and thereby also gain teaching experience. This active learning is unlike anything offered in traditional classrooms, and many students view these classes as a highlight of the program. At other museums, opportunities for specimen preparation could extend to all types of collections, from pinning insects to pressing plants.

### Field opportunities

Undergraduates also have opportunities to participate in regional and international fieldwork. Through such trips, which can provide a transformative experience via active learning, students are exposed to field methods and diverse wildlife (Fig 2). In addition, students learn the practical aspects of fieldwork (e.g., permitting, equipment preparation, trip planning) and can apply their specimen preparation skills. This hands-on field training, combined with the subsequent curation of specimens generated by these trips, instills a comprehensive, experiential understanding of museum practices and specimen-based research. If an institution lacks an extensive field program, opportunities might include taking trips to nearby open spaces, connecting students with local naturalist clubs, or hosting workshops on contributing observations to citizen science database platforms such as eBird or iNaturalist.

## Scientific benefits to the museum

Creating and maintaining a program of this type requires planning, creativity, dedication, and ongoing investment. Ultimately, the results are well worth the effort, as evidenced by the outcomes we describe here. Undergraduates now perform much of the museum’s curatorial activities (Table 1), including preparing and cataloging specimens, digitizing data, and shipping loans to other institutions. Undergraduate involvement is particularly crucial for processing the large numbers of salvaged specimens (Table 1), whose preparation and curation are not supported by external research funding.

**Table 1:** Scientific benefits to the museum. Summary of MVZ curatorial activities performed by undergraduates, both paid and work-for-credit, and their collective output over 10 years (1 June 2005 to 31 May 2015). Because the museum’s specimen data are freely available online (http://arctosdb.org, http://vertnet.org, http://gbif.org, http://www.idigbio.org), these student efforts benefit not just the museum but also the broader scientific community. Data collection details are available in S1 Text.

Activity	Total Number	Percent of Total MVZ Output
Specimens Cataloged	28,323	61%
Loans Shipped	737 (19,070 items)	44%
Skeletons Processed	7,635	99%
Specimens Prepared—Salvaged	3,479 (437 species)	60%
Specimens Prepared—Field	6,801 (6 countries)	23%

MVZ, Museum of Vertebrate Zoology.

Undergraduate participation enriches the broader impacts of grant proposals and helps to attract more funding through increased research productivity. For example, undergraduate field assistants facilitate the collection and curation of an expanded suite of samples (e.g., different tissues for gene expression, blood slides for parasite screening) and associated data (e.g., habitat information, photos), thereby enhancing the value of each specimen. Likewise, undergraduates with molecular lab training help to increase the amount of genetic data captured from museum specimens.

In addition to these practical benefits, undergraduates enhance the vitality of the museum through their intellectual energy by asking questions and contributing ideas, which brings new perspectives to biodiversity science. For example, undergraduates were involved in the conception of AmphibiaWeb (http://amphibiaweb.org), a web portal for amphibian biology, conservation, and taxonomy that has broad benefit to the scientific community and continues to grow due to the contributions of student writers and editors. More generally, undergraduate engagement helps to train the next generation of science, technology, engineering, and math (STEM) researchers and to create well-educated, scientifically literate citizens who can become future advocates for biodiversity and museums.

## Educational benefits to students

The primary benefit to undergraduates of joining a museum-based program is an unparalleled opportunity to work with professional scientists in the process of collections-based research and discovery (Table 2). Students who work with specimens acquire a broader understanding of vertebrate diversity than they would in conventional biology lectures or labs [1,2].

**Table 2:** Educational benefits to students. Summary of primary MVZ positions open to undergraduates, the number of students occupying those positions over 10 years (1 June 2005 to 31 May 2015), and the skills gained in each. Curatorial and preparation lab tasks in particular (row 1) are a natural conduit to research since many of the required skills overlap (e.g., attention to detail, familiarity with specimen data, meticulous record keeping, aptitude for problem solving). Data collection details are available in S1 Text.

Position	Number of Students	Major Skills Gained by Undergraduates
Curatorial & Prep Lab	367	Specimen Data Management & Curation, Specimen Preparation, Museums Loans & Permits, Public Outreach
Molecular Lab	205	DNA Extraction, PCR, Sequencing, Microsatellites
Biodiversity Informatics	54	Georeferencing, Geographic Information Systems (GIS), Museum Databases & Programming, Website Development
Field Assistants	38	Mistnetting, Trapping, Field Notes & Data Collection
Archives	25	Preservation & Digitization of Primary Source Materials
AmphibiaWeb	86	Literature Research, Species Account Writing and Editing, Public Outreach

MVZ, Museum of Vertebrate Zoology.

Undergraduate programs with a tiered structure (Fig 1) allow dedicated students to acquire a reputation for excellence, which in turn can facilitate moving to a higher-level research position within the program (“Advanced” tier, Fig 1) by recommendation from a supervisor. Advanced undergraduates are well positioned to pursue their own research as part of a supervised honors thesis or independent project; since 2005, 32 undergraduates in the MVZ program have co-authored 31 peer-reviewed journal articles (S3 Text), and many more have presented at conferences (Fig 2).

The diverse career paths taken by undergraduates who participate in the museum’s program underscore the broad applicability of their training (S2 Fig). Because these students experience a range of tasks and positions, they gain many valuable skills (Table 2) prior to entering the job market or applying for advanced degrees (S2 Fig). For example, dissections in the preparation lab give aspiring doctors and veterinarians a strong foundation in comparative anatomy, while work in the molecular lab trains students for jobs in biotechnology. Opportunities are not limited to scientific positions [18]. For example, students who work with museum archives are trained in document curation that prepares them for library work, whereas students involved with informatics are equipped for computer science or data management positions.

Finally, an important benefit to students is their immersion in a vibrant research community where they are treated as colleagues, not just trainees (Box 1). Undergraduates in the museum join faculty lab meetings, attend conferences, and participate in workshops. Many students work for multiple supervisors, which has been shown to positively impact the outcomes of participation in research programs [19]. Undergraduates also interact regularly with researchers and faculty in both formal academic settings and informal social venues, which in our experience has helped to break down social barriers within the museum community. Because of these interactions outside a classroom, supervisors can write richer, more detailed letters of recommendation for students. Likewise, faculty have observed that students who work in the museum are more likely to engage enthusiastically in classes. This is consistent with research indicating that undergraduates are more involved in learning when they feel comfortable approaching faculty [20]. The scientific identity and sense of belonging fostered by being part of a community also undoubtedly plays a role in retention; many students remain involved in the museum throughout and beyond their undergraduate careers (S4 Text).

**Box 1. Student stories**

- “[In the MVZ] my first two projects were ‘weeder’ projects, to assess how closely I paid attention to detail. The first was to sort through the reprint collection and alphabetize reprints. I still remembered the authors of those reprints and what the studies were about when I attended my first society meeting, and I was able to hold conversations with established professors as an undergraduate.”–**PhD Student, Evolutionary Biology (4 years in the MVZ)**
- “The MVZ Undergraduate Program helped me to discover my love for the process of science and led me to grad school to continue my exploration. In grad school, I discovered my passion for teaching which led to my career as a high school biology teacher in which I get to engage students in scientific research by teaching them how to DO science the way that the MVZ program taught me.”–**High School Biology Teacher (2 years in the MVZ)**
- “I happened upon fieldwork with the MVZ and have been hooked on birds since. At the MVZ, I found an engaging community of amazing researchers, graduate students, and undergrads just getting their feet wet like me … The love and enthusiasm professors and graduate students had for biodiversity and evolution was what inspired me to become a biologist.”–**Geographic Information System (GIS) Analyst, Environmental Nonprofit (3 years in the MVZ)**
- “Working with the MVZ’s historic field notebooks, photographs and annotated maps introduced me to the joys of interacting directly with historically significant primary sources … It was thrilling to dig through the field notebooks, photographs, and annotated maps of past MVZers, extracting stories that contextualized the museum’s specimens and brought to life the often entertaining and sometimes harrowing experiences of researchers out in the field over the last century.”–**California State Archivist (4 years in the MVZ)**
- “I entered UC Berkeley without any idea of what I wanted to major in, much less what I wanted to go into for a career. The community I found by joining the MVZ Undergraduate Program was with me every step of the way as I explored options ranging from pharmacy to graduate school to finally medical school.”**–Medical Student (3 years in the MVZ)**
- “In the MVZ I remember seeing a walrus skull, a poison dart frog, and a shoebill stork in the span of 15 minutes. The sheer biodiversity contained only within the museum is absolutely astounding, and seeing specimens in the museum as well as in the field creates an incredibly comprehensive learning experience for aspiring biologists.”–**Biologist, Environmental Consulting Firm (1 year in the MVZ)**

## Promoting diversity in science

Research has shown that peer mentoring and active learning improve student performance in STEM courses, especially among undergraduates from groups underrepresented in science [21,22,23]. Furthermore, structured research programs with mentorship are especially effective in promoting the pursuit of advanced degrees among such students [24,25,26]. The tiered design of the MVZ Undergraduate Program, combined with opportunities to earn academic credit or work study, also opens the door for students with no prior experience to join a research program without undue financial or workload burden. These opportunities are critical to recruiting individuals who might otherwise lack the means to participate in unpaid internships [8].

Notably, creating a structured, formalized program has allowed the museum to forge links with other targeted initiatives such as Berkeley’s Biology Scholars Program (BSP, http://bsp.berkeley.edu). BSP is a community support network and mentorship program for undergraduates from groups who face extra barriers to the sciences, including first-generation college students, individuals from economically disadvantaged backgrounds, and students from underrepresented minorities [27]. Such partnerships have facilitated the museum’s efforts to engage a diverse group of students (over the past 10 years, 66 BSP students have participated in the MVZ Undergraduate Program), enriched the museum’s community through inclusion of individuals with different personal and cultural backgrounds, and positively impacted the undergraduate students involved [27].

## Getting started—First steps to program development

For museums interested in developing or strengthening an undergraduate program, we offer five suggestions to increase program effectiveness based on our experiences over the past decade. These low-cost elements are scalable to meet the needs of collections and campuses of different sizes and actively engage students in ways that more isolated, less structured opportunities do not.

### Designate a program coordinator

A coordinator improves logistical oversight and continuity, promotes interactions among participants across disciplines, and facilitates matching student interests to available positions. Additionally, a coordinator can more effectively track students, collect feedback, and encourage prolonged engagement by students and staff. We recognize that many institutions may not have the funds to hire a coordinator, but allocating even part of a person’s time to this role can help facilitate starting an undergraduate program.

### Offer academic credit

Compensation in the form of academic credit provides an official record of a student’s involvement in museum-based training. At the same time, receiving units may allow more students to fit such experiences into their education within a traditional four-year degree program. Furthermore, formally organizing the program by offering academic credit may help to secure further resources to support undergraduate involvement in collections. For institutions that are not affiliated with a university, we recommend offering an official certificate in lieu of credit to formally recognize a student’s work and training.

### Provide diverse opportunities

A program with many opportunities will appeal to a larger and more diverse population of undergraduates (S1 Fig), especially when students can choose their activities. By maximizing exposure to different aspects of museum science and biodiversity research, students are more likely to find the positions that best fit their interests, thus increasing enthusiasm, satisfaction, and, hence, retention.

### Use a tiered structure

A tiered program provides a clear path by which students can progress from entry-level tasks to more skilled, research-focused positions (Fig 1). Students may join the program with no previous experience, thus enabling the museum to recruit freshmen, transfer students, and individuals from disadvantaged or low-income backgrounds. With a tiered structure, relatively mundane initial tasks are viewed as part of a process, not as the endpoint of a student’s experience. A tiered progression also provides a mechanism for evaluating and vetting students for more complex tasks before giving them greater responsibilities.

### Foster a strong community

An engaged community of colleagues and mentors, including peers, promotes relationships that endure far beyond the undergraduate years. For students who are intrigued by biodiversity but initially less interested in research, immersion in a strong, dynamic community can trigger interest in science (Box 1, S4 Text). For all students, a sense of community helps them to feel valued, which increases performance and retention. Efforts to promote community can be as simple as inviting undergraduates to social events and seminars. These approaches are easy to enact and are essential to successfully engaging undergraduates, especially from underrepresented groups [27].

We encourage each organization to be creative and to build upon its resources, academic strengths, and institutional needs to create a program that will be most effective at recruiting, training, and retaining enthusiastic undergraduates.

## Conclusions and future directions

With the development of new technologies and emerging threats to biodiversity, the importance of museum-based research continues to grow [4,28,29,30]. At the same time, support for museums [3,11,12,13,15] and for training in natural history-based biology [6,7] continues to decline. In light of these circumstances, we believe that initiatives like the MVZ Undergraduate Program will increase in importance over time.

Moving forward, we plan to more rigorously evaluate the benefits of this type of biodiversity science-based undergraduate program through formal assessments. For example, use of the Knowledge of Evolution Exam (KEE) [31] will allow us to quantify whether students’ grasp of evolution is improved after working with museum specimens. In addition, improved tracking of undergraduates by developing an alumni database will help us to better understand the long-term impact of the MVZ Undergraduate Program on students’ career paths. Specifically, we are interested in the effects of these experiences on long-term retention in jobs related to the undergraduate experience in the MVZ. We have begun these efforts by developing a more formal network of undergraduate alumni and by hosting the MVZ’s first alumni event in September 2017 (Natural History of the Vertebrates Reunion, https://naturalhistory.berkeley.edu/).

As current museum scientists and wildlife professionals retire [32,33], the resulting void must be filled by a new generation of researchers who are willing to advocate for the relevancy of natural history and museums [7]. Furthermore, although important laws to protect biodiversity have been passed over the last century, with the de-emphasis of natural history education, there are now fewer people trained to implement these regulations [34]. Undergraduates who participate in activities such as the MVZ Undergraduate Program are especially well positioned to fill these needs. Equally important, students who choose other nonbiodiversity careers can, as a result of their experience working in collections, more effectively advocate for museums, conservation, and biodiversity science. No matter what interests students ultimately pursue, undergraduates who join a program like the one described here benefit by working with researchers in a dynamic academic community while gaining real-world exposure to the scientific process.

## Supporting information

S1 Fig Diversity of majors. URAP students (*n* = 384) enrolled in the MVZ Undergraduate Program between 1 June 2005 and 31 May 2015 represented 38 different majors. MVZ, Museum of Vertebrate Zoology; URAP, Undergraduate Research Apprentice Program.
(JPG)

S2 Fig Network diagram. Shows the paths that students take during their training in the MVZ and their subsequent, post-graduation career tracks. Red circles represent MVZ positions, blue circles represent post-graduation positions. Node sizes represent the relative number of individuals engaged in each job. MVZ, Museum of Vertebrate Zoology.
(JPG)

S1 Text Supplementary information on program details and data collection. (DOC)

S2 Text Example museum Undergraduate Program application. (DOC)

S3 Text List of publications with MVZ undergraduate authors. MVZ, Museum of Vertebrate Zoology.
(DOCX)

S4 Text Student stories continued from Box 1. (DOCX)

## Abbreviations

AIM-UP!: Advancing Integration of Museums into Undergraduate Programs
BSP: Biology Scholars Program
CUREs: Course-Based Undergraduate Research Experiences
KEE: Knowledge of Evolution Exam
MVZ: Museum of Vertebrate Zoology
STEM: science, technology, engineering, and math
URAP: Undergraduate Research Apprentice Program
