# Supplementary figures and images for: Mutualism in museums: A model for engaging undergraduates in biodiversity science
Source: PLoS Biol. 2017 Nov 21;15(11):e2003318. doi: 10.1371/journal.pbio.2003318 (PMC5716603; doi:10.1371/journal.pbio.2003318)

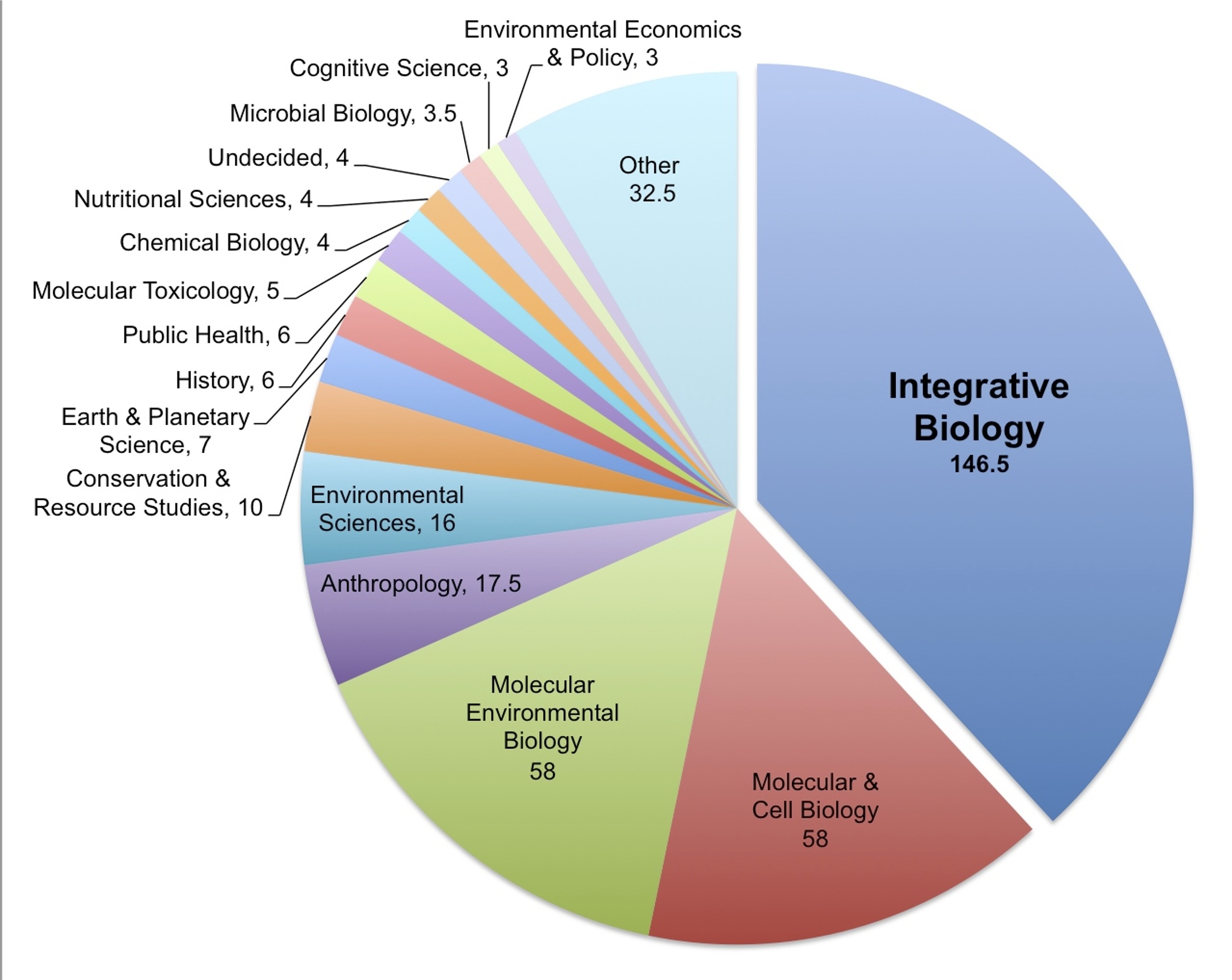

Supplement: S1 Fig — URAP students (n = 384) enrolled in the MVZ Undergraduate Program between 1 June 2005 and 31 May 2015 represented 38 different majors. MVZ, Museum of Vertebrate Zoology; URAP, Undergraduate Research Apprentice Program. (JPG) [file pbio.2003318.s001.jpg]

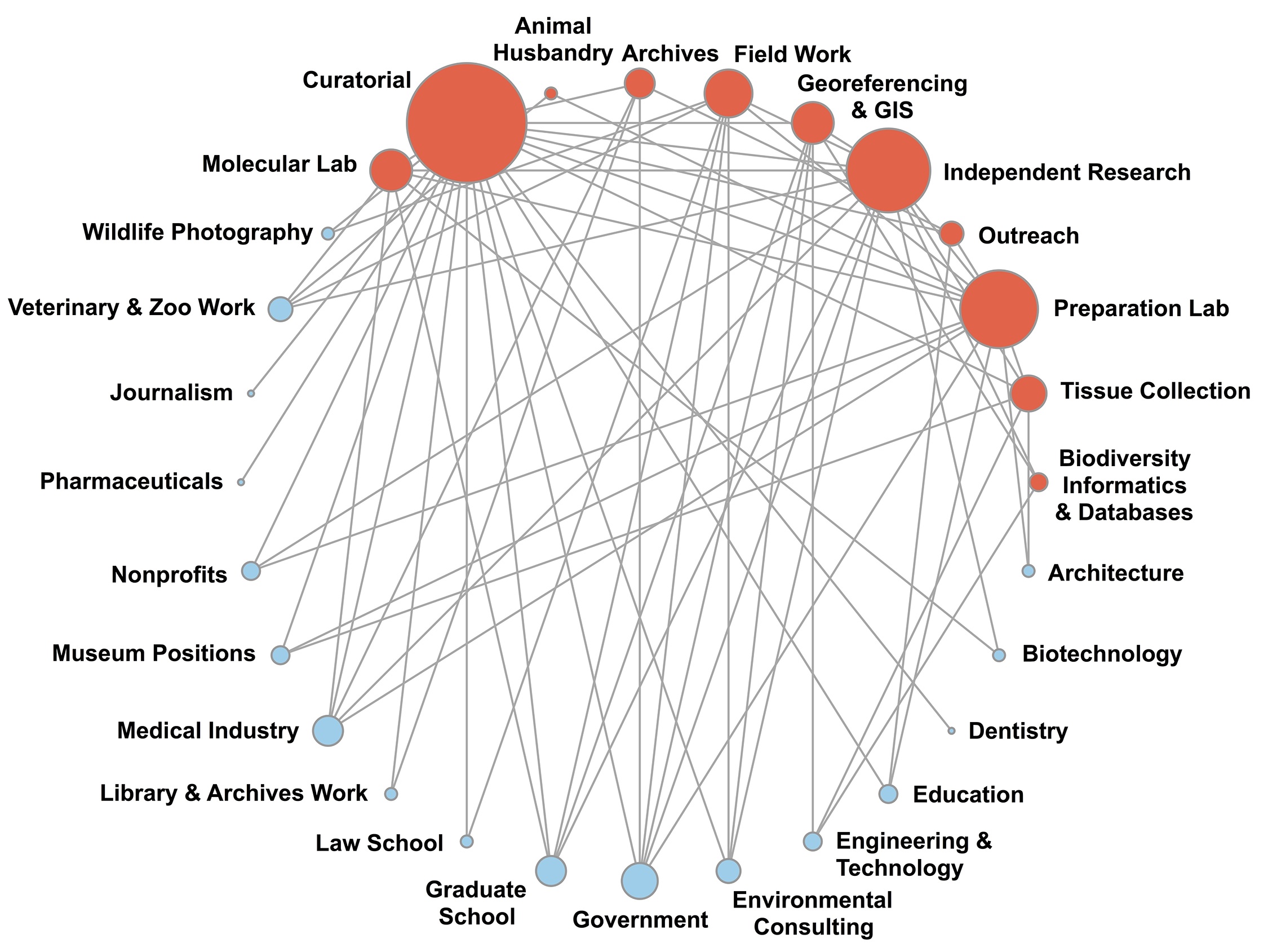

Supplement: S2 Fig — Shows the paths that students take during their training in the MVZ and their subsequent, post-graduation career tracks. Red circles represent MVZ positions, blue circles represent post-graduation positions. Node sizes represent the relative number of individuals engaged in each job. MVZ, Museum of Vertebrate Zoology. (JPG) [file pbio.2003318.s002.jpg]
