# Supplementary material for: Mutualism in museums: A model for engaging undergraduates in biodiversity science
Source: PLoS Biol. 2017 Nov 21;15(11):e2003318. doi: 10.1371/journal.pbio.2003318 (PMC5716603; doi:10.1371/journal.pbio.2003318)
Supplement: S1 Text — (DOC) [file pbio.2003318.s003.doc]

**Additional Program Details**

Additional program details can be found online (below). Specific materials used in the MVZ Undergraduate Program (e.g., preparation training guides, curatorial training guides, evaluation forms) are available from the authors of this article upon request.

Aimed at Students:

<http://mvz.berkeley.edu/Undergrad_opps.php>

Aimed at Supervisors:

<http://mvz.berkeley.edu/MVZ_ForUG-VolSups.php>

**Supplementary Information on Data Collection**

**Table 1:**

Unless otherwise noted, records are from 1 June 2005 to 31 May 2015. Data were obtained from the Arctos database ([http://arctosdb.org](http://arctosdb.org/)).

Specimens Cataloged- Percentage is based on specimens cataloged by faculty, staff, graduate students, and undergraduates. If faculty and staff are removed, the specimens cataloged by MVZ undergraduates rises to 87% of the total. Curators vet and review all data for quality control.

Loans Shipped- Arctos tracks loan activity through shipments, and thus numbers only reflect loans packed by undergraduates. However, most loans involve undergraduate help (e.g., tissue subsampling, retrieving specimens from the collections). The taxonomic breakdown of these loans (N =737) was 20% avian, 27% mammalian, and 53% herpetological.

Skeletons Processed- Numbers are from January 2008 through 2015; detailed records were not kept prior to 2008.

Specimens Prepared, Field - Total is based on undergraduate student names associated with field-collected specimens in the Arctos database. This number represents specimens for which undergraduates either assisted in preparation or prepared the entire specimen themselves. Countries represented include Ghana, Guatemala, Indonesia, French Polynesia, Mexico, and the United States.

**Table 2:**

The data reflect student participation from 1 June 2005 to 31 May 2015, unless otherwise noted.

Curatorial & Prep Lab- Many MVZ undergraduates participated in both curatorial and specimen preparation activities. Of the 367 total students in this combined category, 307 prepared salvaged specimens, 40 packed specimen loans, and 39 cataloged specimens (Table 1).

Molecular Lab- Includes all undergraduates who worked in the MVZ-affiliated Evolutionary Genetics Lab (EGL) since 2007. This number includes students in the MVZ Undergraduate Program as well as those recruited directly as research assistants outside of the program. Approximately 58% of the EGL users between 2007-2015 were undergraduates.

Archives- Undergraduate contributions to the MVZ Archives began in 2013.

**S1 Figure**, Diversity of Majors:

Although the MVZ is most closely associated with the Department of Integrative Biology, 62% of URAP students declared majors in other departments, further demonstrating the diversity of individuals engaged by a museum-based program. The “Other” category includes 22 majors with fewer than three students represented. Students with double majors were counted as 0.5 in each major.

**Disclaimer on Human Subject Research**

This work was not subject to human subjects research (HSR) regulations because we conducted this review as a single case study to evaluate and measure the effectiveness of an individual program. We did not attempt to compare to other programs nor were data analyzed to test a hypothesis. No individually identifying personal information was used in this publication.

All individuals in Fig 2 provided written consent for us to publish their photos for this article.
