# Supplementary material for: Mutualism in museums: A model for engaging undergraduates in biodiversity science
Source: PLoS Biol. 2017 Nov 21;15(11):e2003318. doi: 10.1371/journal.pbio.2003318 (PMC5716603; doi:10.1371/journal.pbio.2003318)
Supplement: S2 Text — (DOC) [file pbio.2003318.s004.doc]

**Museum Undergraduate Application**

By filling out this form, you provide us with the tools to properly place you in positions that you will find rewarding. We may also use it as a reference so that we can recommend you for potential volunteer, research, or job opportunities here or elsewhere in the future.

Name: ______________________________________ Email: ______________________________________

Today's date (day month year): ______________________

Current student status: _____________________________ GPA: ____________

Major or intended major: ____________________________________________
What year and semester do you expect to graduate? ________________________

What are your interests that relate to museums and biology? (evolution, behavior, pathology, biodiversity, species distributions, etc.)

Do you have a specific career that you are pursuing, if so what is it?

Which of the following would you be interested in?:

___ Volunteer positions ___ Paid positions

___ Spring semester opportunities ___ Summer opportunities

___ Fall semester opportunities ___ Positions for academic credit

Are you eligible for work-study? _____________

Are you currently in a museum position, or are you applying for one?

___ I am currently in a museum position.

___ I am applying for a museum position.

What is the position you are currently in/applying for? ____________________________________________

Supervisor: _______________________________

Have you had past involvement in the museum?

If so, doing what?__________________________________________________________________________

Supervisor:________________________________

**Your Interests (as they relate to the museum)**

Would you be interested in working in specimen preparation? (this entails animal necropsy and specimen dissection): ___________
If yes, do you have previous experience with animal dissection and/or preparation? (please provide a brief explanation): ___________

Would you be interested in being involved in museum archival work/historical projects? ___________

Would you be interested in being involved in GIS/GPS mapping work (georeferencing)? ___________

Would you be interested in being involved in the molecular genetics lab? ___________
Do you have genetics lab experience? ___________
If the answer is yes, please give a one or two sentence summary of what you worked on:

Would you be interested in being involved in fieldwork? ___________
Do you have fieldwork experience? ___________
If the answer is yes, please give a one or two sentence summary of what you were involved in:

Would you be interested in being involved in museum outreach and education work? ___________
If yes, which of the following would you be interested in?:

___ Creating displays ___ Making posters

___ Tours ___ Outreach to classrooms

___ Website development ___ Scientific writing
If the answer is yes to website development, do you have HTML, Dreamweaver or other relevant experience? (please explain):___________________________________________________________________________

If the answer is yes to making posters, do you have experience in Adobe Photoshop, Illustrator, or other design programs? (please explain):__________________________________________________________________
Which of the following opportunities would you be interested in getting involved in?:

___ Collections research ___ Morphological research ___ Animal lab care

___ Biomechanics research ___ Library research ___ Museum curatorial work

___ Animal behavior research ___ Scientific illustration ___ Museum collections maintenance

___ GIS (Spatial data, analysis and mapping)

Are you looking for a senior thesis project for this year or next year? _________________________________

**Other Questions**

How did you hear about the museum (choose one or more of the following):

___ Museum website

___ Other website (specify): ____________________________________

___ Museum Tour

___ Public Event

___ Other (specify): ___________________________________________

What classes have you taken that would be relevant to your position, or possible future positions within the museum? (please note semester taken)

What are you hoping to get out of your experience in the museum?

Do you have any other previous experience that you would like us to know about?

Anything else you'd like us to be aware of?

Please write the numbers 0 – 9 in your smallest and neatest handwriting on the following line:

_______________________________________________

**Thank you for taking the time to fill this out!**

Please hand this form in to the front office of the museum, address:
