# Supplementary material for: Mutualism in museums: A model for engaging undergraduates in biodiversity science
Source: PLoS Biol. 2017 Nov 21;15(11):e2003318. doi: 10.1371/journal.pbio.2003318 (PMC5716603; doi:10.1371/journal.pbio.2003318)
Supplement: S3 Text — MVZ, Museum of Vertebrate Zoology. (DOCX) [file pbio.2003318.s005.docx]

**MVZ Undergraduate Publications**

Peer-reviewed publications from the museum including an MVZ undergraduate author (in bold). Additional publications are still in preparation or in review.

1. **Bell, R. C.**, J. L. Parra, **M. Tonione**, C. J. Hoskin, J. B. MacKenzie, S. E. Williams, and C. Moritz. 2010. Patterns of persistence and isolation indicate resilience to climate change in montane rainforest lizards. *Molecular Ecology,* 19:2531-2544.

2. Bi, K., **T. Linderoth**, D. Vanderpool, J. M. Good, R. Nielsen, and C. Moritz. 2013. Unlocking the vault: next-generation museum population genomics. *Molecular Ecology,* 22:6018-6032.

3. **Bouzid, N. M.**, S. M. Rovito, and J. F. Sanchez-Solís. 2015. Discovery of the critically endangered Finca Chiblac Salamander (*Bradytriton silus*) in northern Chiapas, Mexico. *Herpetological Review,* 46:186-187.

4. Bowie, R. C. K., K. A. Feldheim, **Z. R. Hanna**, A. B. Sellas, and C. Cicero. 2017. Development of polymorphic tetranucleotide microsatellite markers for New World Warblers (Aves: Passeriformes: Parulidae) with broad cross-species utility. *Wilson Bulletin,* 129:401-407.

5. **Chong, R. A.** and J. A. McGuire. 2008. Geographic distribution: *Psammodynastes pictus*. *Herpetological Review,* 39:112.

6. Clark, C. J. and **T. J. Feo**. 2008. The Anna's hummingbird chirps with its tail: a new mechanism of sonation in birds. *Proceedings of the Royal Society of London B: Biological Sciences,* 275:955-962.

7. Clark, C. J. and **T. J. Feo**. 2009. Why do Calypte hummingbirds “sing” with both their tail and their syrinx? An apparent example of sexual sensory bias. *The American Naturalist,* 175:27-37.

8. Conroy, C. J., J. L. Patton, **M. C.** **Lim**, **M. A.** **Phuong**, B. Parmenter, and S. Höhna. 2016. Following the rivers: historical reconstruction of California voles *Microtus californicus* (Rodentia: Cricetidae) in the deserts of eastern California. *Biological Journal of the Linnean Society*, 119:80-98.

9. **Eastman, L. M.**, T. L. Morelli, K. C. Rowe, C. J. Conroy, and C. Moritz. 2012. Size increase in high elevation ground squirrels over the last century. *Global Change Biology,* 18:1499-1508.

10. **Feo, T. J.** and C. J. Clark. 2010. The displays and sonations of the Black-chinned Hummingbird (Trochilidae: *Archilochus alexandri*). *The Auk,* 127:787-796.

11. **Hiller, A. E.**, C. Cicero, **M. J. Albe**, **T. L. Wong**, C. Spencer, R. C. K. Bowie, and E. A. Lacey. In review. Mutualism in museums: A model for engaging undergraduates in biodiversity science. *PLOS Biology*.

12. **Karin B. R.**, **A. L. Stubbs**, and U. Arifin. 2013. *Limnonectes grunniens* Saline Water Tolerance. *Herpetological Review,* 44:656-656.

13. Leaché, A. D., **R. A. Chong**, T. J. Papenfuss, P. Wagner, W. Böhme, A. Schmitz, M. O. Rödel, M. LeBreton, I. Ineich, L. Chirio, A. Bauer, E. A. Eniang, and S. Baha El Din. 2009. Phylogeny of the genus *Agama* based on mitochondrial DNA sequence data. *Bonner zoologische Beiträge*, 56:273-278.

14. Leaché, A. L., **D. Helmer**, and C. Moritz. 2010. Phenotypic evolution in high-elevation populations of western fence lizards (*Sceloporus occidentalis*) in the Sierra Nevada Mountains. *Biological Journal of the Linnean Society*, 100:630-641.

15. Maher, S. P., T. L. Morelli, **M.** **Hershey**, A. L. Flint, L. E. Flint, C. Moritz, and S. R. Beissinger. 2017. Erosion of refugia in the Sierra Nevada meadows network with climate change. *Ecosphere*, 8:4.

16. McEntee, J. P., **J. V. Peñalba**, C. Werema, E. Mulungu, M. Mbilinyi, D. Moyer, L. Hansen, J. Fjeldså, and R. C. K. Bowie. 2016. Social selection parapatry in Afrotropical sunbirds. *Evolution*, 70:1307-1321.

17. Morelli, T. L., A. B. Smith, **C. R. Kastely**, **I. Mastroserio**, C. Moritz, and S. R. Beissinger. 2012. Anthropogenic refugia ameliorate the severe climate-related decline of a montane mammal along its trailing edge. *Proceedings of the Royal Society of London B: Biological Sciences*, 279:4279-4286.

18**. Oza, A. U.**, **K. E. Lovett**, S. E. Williams, and C. Moritz. 2012. Recent speciation and limited phylogeographic structure in *Mixophyes* frogs from the Australian Wet Tropics. *Molecular phylogenetics and evolution*, 62:407-413.

19. Parmenter, B.E., **M.C.W. Lim**, Y. Chen, C.J. Conroy, and B.F.M. Olechnowski. 2015. Microsatellite variation in the Owens Valley vole: *Microtus californicus vallicola*. *Southwestern Naturalist*, 60:256-262.

20. **Peñalba J. V.**, L. L. Smith, M. A. Tonione, C. Sass, S. M. Hykin, P. L. Skipwith, J. A. McGuire, R. C. K. Bowie, and C. Moritz. 2014. Sequence capture using PCR-generated probes: a cost-effective method of targeted high-throughput sequencing for nonmodel organisms. *Molecular Ecology Resources*, 14:1000-1010.

21. **Phuong M. A.**, **M. C. W. Lim**, **D. R. Wait**, K. C. Rowe, and C. Moritz. 2014. Delimiting species in the genus *Otospermophilus* (Rodentia: Sciuridae), using genetics, ecology, and morphology. *Biological Journal of the Linnean Society*, 113:1136-1151.

22. **Phuong, M. A.**, K. Bi, and C. Moritz. 2017. Range instability leads to cytonuclear discordance in a morphologically cryptic ground squirrel species complex. *Molecular ecology*.

21. Rubidge, E. M., J. L. Patton, **M. Lim**, A. C. Burton, J. S. Brashares, and C. Moritz. 2012. Climate-induced range contraction drives genetic erosion in an alpine mammal. *Nature Climate Change*, 2:285-288.

22. **Shohfi, H. E.**, C. J. Conroy, A. R. Wilhelm AR, and J. L. Patton. 2006. New records of *Sorex preblei* and *S. tenellus* in California. *The Southwestern Naturalist*, 51:108-11.

23. **Shultz, A. J.**, M. W. Tingley, and R. C. K. Bowie. 2012. A century of avian community turnover in an urban green space in northern California. *Condor*, 114:258-267.

24. Snyder, H. K., R. Maia, L. D'Alba, **A. J.** **Shultz**, K. M. Rowe, K. C. Rowe, and M. D. Shawkey. 2012. Iridescent colour production in hairs of blind golden moles (Chrysochloridae). *Biology letters*, 8:393-396.

25. Swei, A, J. J. L. Rowley, D. Rödder, M. L. L. Diesmos, A. C. Diesmos, C. J. Briggs, R. M. Brown, T. T. Cao, T. L. Cheng, **R. A. Chong**, B. Han, J.-M. Hero, H. D. Hoang, M. D. Kusrini, D. T. T. Le, J. A. McGuire, M. Meegaskumbura, M.-S. Min, D. G. Mulcahy, T. Neang, S. Phimmachak, D.-Q. Rao, **N. M. Reeder**, S. D. Schoville, N. Sivongxay, N. Srei, M. Stöck, B. L. Stuart, L. S. Torres, D. T. A. Tran, T. S. Tunstall, D. Vieites, and V. T. Vredenburg. 2011. Is Chytridiomycosis an Emerging Infectious Disease in Asia? *PLoS* ONE, 6:e23179.

26. Tomiya, S., J. L. McGuire, R. W. Dedon, S. D. Lerner, **R. Setsuda**, **A. N. Lipps**, J. F. Bailey, K. R. Hale, A. B. Shabel, and A. D. Barnosky. 2011. A report on late Quaternary vertebrate fossil assemblages from the eastern San Francisco Bay region, California. *PaleoBios*, 30:2.

27. Voelker, G., J. W. Huntley, **J. V. Peñalba**, and R. C. K. Bowie. 2016. Resolving taxonomic uncertainty and historical biogeographic patterns in Muscicapa flycatchers and their allies. *Molecular Phylogenetics and Evolution*, 94:618-625.

28. Voelker G., **J. V. Peñalba**, J. W. Huntley, and R. C. K. Bowie. 2014. Diversification in an Afro-Asian songbird clade (Erythropygia-Copsychus) reveals founder-event speciation via trans-oceanic dispersals and a southern to northern colonization pattern in Africa. *Molecular Phylogenetics and Evolution*, 73:97-105.

29. **Weinstein, S. B.** 2009. An aquatic disease on a terrestrial salamander: Individual and population level effects of the amphibian chytrid fungus, *Batrachochytrium dendrobatidis*, on *Batrachoseps attenuatus* (Plethodontidae). *Copeia*, 2009:653-660.

30. Wogan, G. O., K. A. Feldheim, A. S. Tsai, **A. A.** **Brown, J.** **Kapelke,** M. Galinato, J. N. Tung, J. M. Bates, P. Kaliba, G. Voelker, and R. C. Bowie. 2016. New genetic resources and a preliminary multi-locus assessment of species boundaries in the *Batis capensis* species complex (Passeriformes: Platysteridae). *Biochemical Systematics and Ecology*, 65:83-88.

31. **Yang, D.**, C. J. Conroy, and C. Moritz. 2011. Contrasting responses of *Peromyscus* mice of Yosemite National Park to recent climate change. *Global Change Biology*, 17:2559-2566.
