# Supplementary material for: Mutualism in museums: A model for engaging undergraduates in biodiversity science
Source: PLoS Biol. 2017 Nov 21;15(11):e2003318. doi: 10.1371/journal.pbio.2003318 (PMC5716603; doi:10.1371/journal.pbio.2003318)
Supplement: S4 Text — (DOCX) [file pbio.2003318.s006.docx]

**Student Stories (Continued from Box 1)**

We asked seventeen former students, who represent a diversity of careers, six questions (below) regarding their individual experiences in the museum undergraduate program, receiving responses from all seventeen.

These students represent those with whom we are still in contact thus they are not a random sample, but we hope it adds weight to this work to have students describe their experiences at the MVZ in their own words.

While these responses (selected quotes presented below) were not collected in a standardized way, they provide illustrative examples of how undergraduates can benefit from involvement in a museum-based program.

**Questions**

(1) Provide your name, current affiliation/title, years in the MVZ (date range)

(2) How did the MVZ Undergraduate Program contribute uniquely to your understanding and appreciation of biodiversity, ecology, evolution, and/or conservation?

(3) How did the MVZ Undergraduate Program provide a transformative experience during your undergraduate years at UCB?

(4) How did being part of the MVZ community influence your interest in science and research?

(5) How did the MVZ Undergraduate Program fit into your overall undergraduate experience and career path?

(6) Provide an example of an active or hands-on learning experience that was particularly memorable (e.g., prep lab, field work), and what you got out of it.

**Selected Responses**

(Number of years at the MVZ in parentheses)

1. GIS analyst and biologist, environmental non-profit. (3)

“The MVZ Undergraduate Program was the single most important experience that got me into the field of avian ecology and conservation. The love and enthusiasm professors and graduate students had for biodiversity and evolution was what inspired me to become a biologist!

I’ll never forget the day we were mist-netting in the Lassen transect and caught hundreds of Yellow-rumped Warblers in a set of nets. It was all-hands-on-deck and we quickly shifted focus from carefully documenting every single bird to just extract as fast as you can […] and let them go! It was definitely a rush and bonding experience, not to mention incredible practice in extraction.”

2. PhD student, evolutionary biology and natural history museums. (4)

“Joining the MVZ Undergraduate Program provided me with a network of academic and social role models. I had concrete examples of the different stages of an academic career. My time at MVZ played a huge part in my individual development and helped me feel like I belonged at UC Berkeley.

My first two projects were “weeder” projects, to assess how closely I paid attention to detail. The first was to sort through the reprint collection and alphabetize reprints. I still remembered the authors of those reprints and what the studies were about when I attended my first society meeting, and I was able to hold conversations with established professors as an undergraduate. My second project was to sort through thousands of tiny, seemingly indistinguishable salamanders (*Batrachoseps*). This project taught me to always double-check catalog numbers and connected me with […] my future undergraduate research advisor.”

3. PhD student, environmental sciences. (5)

“The MVZ actually provided my first opportunity to have hands on research in ecology and biodiversity. The museum did an amazing job showing all the perspectives of ecological research including fieldwork, genetics, and the collections.

The MVZ was integral to my undergraduate experience and career path. It gave me a sense of community, which was incredibly important to feeling like I belonged in science and could make a career out of it in the future.”

4. Medical student. (3)

“The program introduced me to lifelong mentors and friends, without whom I would not be where I am today. Even now, three years out of undergrad, I turn to these individuals for advice on my aspirations. Finally, besides giving me a far deeper understanding of ecology, evolution, and biodiversity, the program was important in helping me grow as an individual – in learning to be more confident, curious, and assertive.

I entered UC Berkeley without any idea of what I wanted to major in, much less what I wanted to go into for a career. The community I found by joining the MVZ Undergraduate Program was with me every step of the way as I explored options ranging from pharmacy to graduate school to finally medical school. As mentioned already, the MVZ allowed me to explore a broad range of scientific interests and allowed me to learn how to effectively teach other students.”

5. State archivist. (7)

“My time at the MVZ was a significant part of my undergraduate experience and directly influenced the trajectory of my professional interests and ultimate career path. The time I spent working with the MVZ’s historic field notebooks, photographs and annotated maps introduced me to the joys of interacting directly with historically significant primary sources and showed me the importance of preserving and providing access to such materials for current and future generations.

As part of my work with the MVZ’s archival materials, I had the opportunity to develop small historical exhibits for Cal Day. It was thrilling to dig through the field notebooks, photographs, and annotated maps of past MVZers, extracting stories that contextualized the museum’s specimens and brought to life the often entertaining and sometimes harrowing experiences of researchers out in the field over the last century.”

6. Manager, tech industry. (3)

“I worked within the GIS department and have found maps to be a very intuitive way for me to understand information. Seeing biodiversity data displayed spatially helped me better understand how tied evolution can be to geographic factors.

I had an interest in programming and geospatial data when I first started at UCB. The MVZ Undergraduate Program provided me with an opportunity to nurture and develop these skills that I believe helped me land my job. I could not have made it to where I am without the amazing mentorship and guidance I’d received while working within the MVZ Undergraduate Program.”

7. Veterinary medicine student. (5)

“[The MVZ Undergraduate Program] made it real! The collection and its history, the field time, the new accessions, and the people working to grow it–being in the museum taught me first-hand the importance of research collections and how historical data allows us to study ecological change.

Prep lab, teaching prep lab, the lab work and fieldwork showed me the value of collaborative learning and about the thrill of pursuing curiosity. Lifelong friends and infinite source of renewed inspiration as I follow what great things they take on next!”

8. Law student. (4)

“Being part of the MVZ made me more aware of the breadth of what can be studied and discovered. I especially enjoyed going to Wednesday coffees and the lunch talks to hear about what everyone was accomplishing!

The MVZ Undergraduate Program ended up being a huge part of my undergraduate experience—I spent much of my time in the MVZ and many of my good friends worked there as well. It also introduced me to fieldwork, which I ended up doing for five years after I graduated.”

9. Biologist, environmental consulting. (1)

“Working at the MVZ helped me appreciate the immense value that museums provide in biological research. Study skins, skeletons, and DNA samples are some of the most valuable and consistent datasets that biologists across the globe can tap into through museums like the MVZ.

The MVZ gave me a community of people who really opened my eyes to the different careers I could pursue in wildlife biology. Having so many grad students, professors, and curators to give me advice and point me in the right direction truly got me where I am today.

In the MVZ I remember seeing a walrus skull, a poison dart frog, and a shoebill stork in the span of 15 minutes. The sheer biodiversity contained only within the museum is absolutely astounding, and seeing specimens in the museum as well as in the field creates an incredibly comprehensive learning experience for aspiring biologists.”

10. Master of architecture student. (4)

“The MVZ was definitely the #1 highlight of UCB. While it was not directly related to my classes, it was still a great experience to apply in a liberal arts degree. Because I was able to bring concepts from the MVZ into my design classes (even as of now) where I apply conservational methods and the importance of biodiversity into my designs. And this was always much more interesting than just the regular design fundamentals.

It’s really interesting when I say I got a biology degree from UC Berkeley, and now I am a Master of Architecture Candidate. People always ask me how it’s relatable, and to me, it’s 100% relatable. How can you be a good designer without knowing the design’s context? I’m still proud until this day to say that I am both a designer and a biologist.”

11. Engineer. (2)

“My computer programmer position with the MVZ was my first technical job and gave me exposure to the tech world, which confirmed to me that a tech career was definitely the right one for me. Not only was the job itself interesting, but it was great to be part of the MVZ community, a tight-knit group of passionate people who care about research in biological diversity.”

12. PhD student, evolutionary biology and natural history museums. (4)

“The MVZ Undergraduate program defined my appreciation for natural history museums as an invaluable for both the research community and science outreach. The program has also motivated me to take a more integrative approach in answering research questions incorporating aspects such as natural history which is often overlooked in many studies.

The unique experience working in the prep lab and within the collections provided me with an appreciation for the rigor involved in preserving, cataloging, and managing an active museum collection, which translates to how I currently collect samples and meta-data for my own research. Being established within a museum sets this program apart from any other lab or field based research programs, as it changes your perspective from collecting samples and data for your own short-term research benefit to longer term and for a broader scientific community.”

13. Pharmaceutical specialist. (3)

“Before I joined MVZ, I was mostly interested in large mammals because they are what I saw at zoos and nature shows. Working hands-on in the collections and prep lab and participating in fieldwork expanded my appreciation for all organisms.

Although I did not end up in the research field, the importance of collecting and preserving data is a big part of my current job. What I learned and experienced from working at the MVZ is very applicable to my field in pharmaceuticals.”

14. Government biologist. (3)

“Intense field and taxonomy classes instilled me with a knowledge of California’s fauna that I still rely on daily in my profession. Working on cutting edge ecological and evolutionary research showed me how the biological sciences are practiced every day, the passion MVZ faculty, students, and staff have for their field, and the impact their efforts make.

Each person in the MVZ is working on something about which they are passionate and that will create new knowledge. I haven’t wanted to do anything else after experiencing that.”

15. High school teacher. (2)

“Through IB 104 and my MVZ Undergraduate Research, I was able to learn how to ask my own questions and figure out how to answer them and really DO science for the first time in my life. Once I was given the opportunity, I felt like I had been kept in the dark about the true nature of science by my previous science courses.

Being a part of the MVZ community was instrumental in developing the skills and the confidence necessary to succeed in science. I felt like I was a part of a family which truly made the intimidating aspects of science-learning new skills, taking risks, and the possibility of failure-much less scary because there was always someone there to ask for help.

The MVZ Undergraduate Program helped me to discover my love for the process of science and led me to grad school to continue my exploration. In grad school, I discovered my passion for teaching which led to my career as a high school biology teacher in which I get to engage students in scientific research by teaching them how to DO science the way that the MVZ program taught me.”

16. Photographer. (7)

“The most important aspect of my time in the MVZ Undergraduate Program was the connections it facilitated. I am still collaborating with researchers I met at the MVZ 10 years ago.

The Undergraduate Program helped me understand what fieldwork and lab work for a research scientist was like in a way that my coursework in integrative biology did not. It was a very useful bridge between my coursework and my current career in science communication.

One of my memorable experiences was my time as a volunteer in the Grinnell resurvey project in Lassen National Park in 2007. I remember the 5am start times to open mist nets and preparing specimens well after sunset. It gave me a sense for the dedication it takes to be a field biologist.”

17. Current undergraduate student. (4)

“The atmosphere the museum staff creates is both calming and energetic that you don’t feel like you’re working but instead indulging in a hobby. It reaffirms ones goal in life to “love what you do and do what you love” which is in this case—animal guts and research papers.

The MVZ gave me a powerful connection to the Berkeley campus and community thereby shaping the experiences of my undergraduate academic career. My classes make more sense and hold more meaning thanks to everything I learn at the MVZ.”
